# Advanced Trauma Care With Tricalcium Phosphate Bone Grafts for Tibial Plateau Fractures: A Report of Three Cases

**DOI:** 10.7759/cureus.74982

**Published:** 2024-12-02

**Authors:** Bárbara Costa, Diogo Pascoal, Ricardo Sousa, Raquel Ricardo, António Figueiredo

**Affiliations:** 1 Orthopedics and Traumatology, Unidade Local de Saúde da Cova da Beira, Covilhã, PRT

**Keywords:** biocompatible materials, bone graft substitutes, open reduction and internal fixation (orif), tibial plateau fracture, tricalcium phosphate

## Abstract

Advances in implants and biological therapies have significantly improved trauma care, offering surgeons a variety of solutions for complex cases. This study evaluates the outcomes of three patients with complex Schatzker type V tibial plateau fractures, treated with open reduction and internal fixation supplemented by tricalcium phosphate bone void filler. The surgical approach was selected based on the specific fracture pattern. Postoperative outcomes were assessed using the Oxford Knee Score (OKS), Visual Analog Scale (VAS) for pain, and EQ-5D-5L for quality of life. The results indicated high patient satisfaction (mean score of 8.67 ± 2.31) and low pain levels (mean VAS of 2.67 ± 2.31). The mean OKS was 32 (± 8.66), and the mean EQ-5D-5L VAS score was 88.33 (± 16.07). Follow-up assessments revealed a good to excellent range of motion, with no significant complications. No additional articular step-off was observed at follow-up. The study suggests that tricalcium phosphate bone grafts may be a promising biomaterial for enhancing recovery in complex tibial plateau fractures by providing mechanical support and promoting osteointegration.

## Introduction

Advances in implants and, more recently, biological therapies have greatly contributed to the evolution of trauma care, equipping trauma surgeons with an expanded array of solutions to address complex and challenging cases. Surgical techniques have continuously evolved, incorporating both technical and biological advancements.

According to the diamond concept described by Giannoudis et al. [1], successful fracture healing requires a favorable local environment, which includes osteogenic cells, osteoinductive mediators, an osteoconductive matrix, adequate mechanical stability, and sufficient blood supply. This concept helps clinicians assess the essential elements for effective fracture healing [1]. Arguably, one of the most critical local factors influencing bone healing is the energy transferred by the injury, including the severity of soft tissue damage [2].

Failure of implant fixation remains a significant concern in orthopedic trauma. Although this complication in tibial plateau fractures has received limited attention in the published literature, Ali et al. identified statistically significant associations with factors such as age over 60 years, premature weight-bearing, preoperative fracture displacement, fragmentation, and severe osteoporosis [3]. In fact, bone mineral density is one of the key factors affecting the success of fracture fixation [4]. In younger patients, tibial plateau fractures are often associated with high-energy trauma, which frequently leads to comminuted fractures. Both low-energy trauma in osteopenic bone and high-energy trauma in younger patients can result in cancellous bone defects and dead space at the fracture site, where the fragments are impacted into the soft metaphyseal bone [5].

When a critical-sized bone defect is present - usually due to bone loss at the time of injury - bone healing and regeneration become significantly more challenging [6]. Therefore, the use of an osteoconductive matrix, along with osteogenic and osteoinductive elements, may be necessary [6].

Bone void fillers are valuable adjuvants when structural grafts are unavailable [7]. These biodegradable calcium-based synthetic materials provide an excellent osteoconductive scaffold for mesenchymal stem cell migration, allow for blood vessel ingrowth, and stimulate new bone formation at rates comparable to autogenous bone [6].

This case report aims to evaluate the outcomes following surgical treatment of three complex tibial plateau fractures using AdvanCore^® ^(Artur Salgado SA, Maia, Portugal) as a bone void filling adjuvant. It will assess functional outcomes and patient satisfaction while describing the surgical technique used for its application.

## Case presentation

We conducted a retrospective review of three patients with complex tibial plateau fractures (Schatzker type V) who underwent surgical treatment with open reduction and internal fixation (ORIF), along with augmentation using tricalcium phosphate bone filler (AdvanCore^®^, Artur Salgado SA). The patients were treated in our department between January 2023 and June 2024. The diagnosis was made based on the results of plain radiographs in the AP and lateral views. Additionally, a CT scan was performed to enhance the determination of the location and extent of the depressed articular surface.

Institutional electronic charts were used for data collection, including basic demographic information, related injuries, treatment choices, and associated complications. All patients presented with tibial plateau depression. Results were obtained through phone interviews conducted by an independent surgeon, using validated scales such as patient satisfaction (on a scale of 0-10), Visual Analog Scale (VAS) score for pain intensity, the Oxford Knee Score (OKS), and EQ-5D-5L. The OKS and EQ-5D-5L used in this study were previously validated for the Portuguese language [8-10].

The OKS consists of 12 questions, with each question scored from zero to four, where zero indicates the worst possible outcome and four indicates the best. After the patient answers each question, the individual scores are summed to yield an overall score ranging from zero (worst outcome) to 48 (best outcome).

The EQ-5D-5L includes five questions on mobility, self-care, usual activities, pain/discomfort, and anxiety/depression. Each question uses a Likert scale. Additionally, there is a section with a VAS (EQ-VAS) where patients rate their own health from 0 to 100, with 0 representing the worst possible health and 100 representing the best possible health.

Characterization of patients

**Figure 1:**
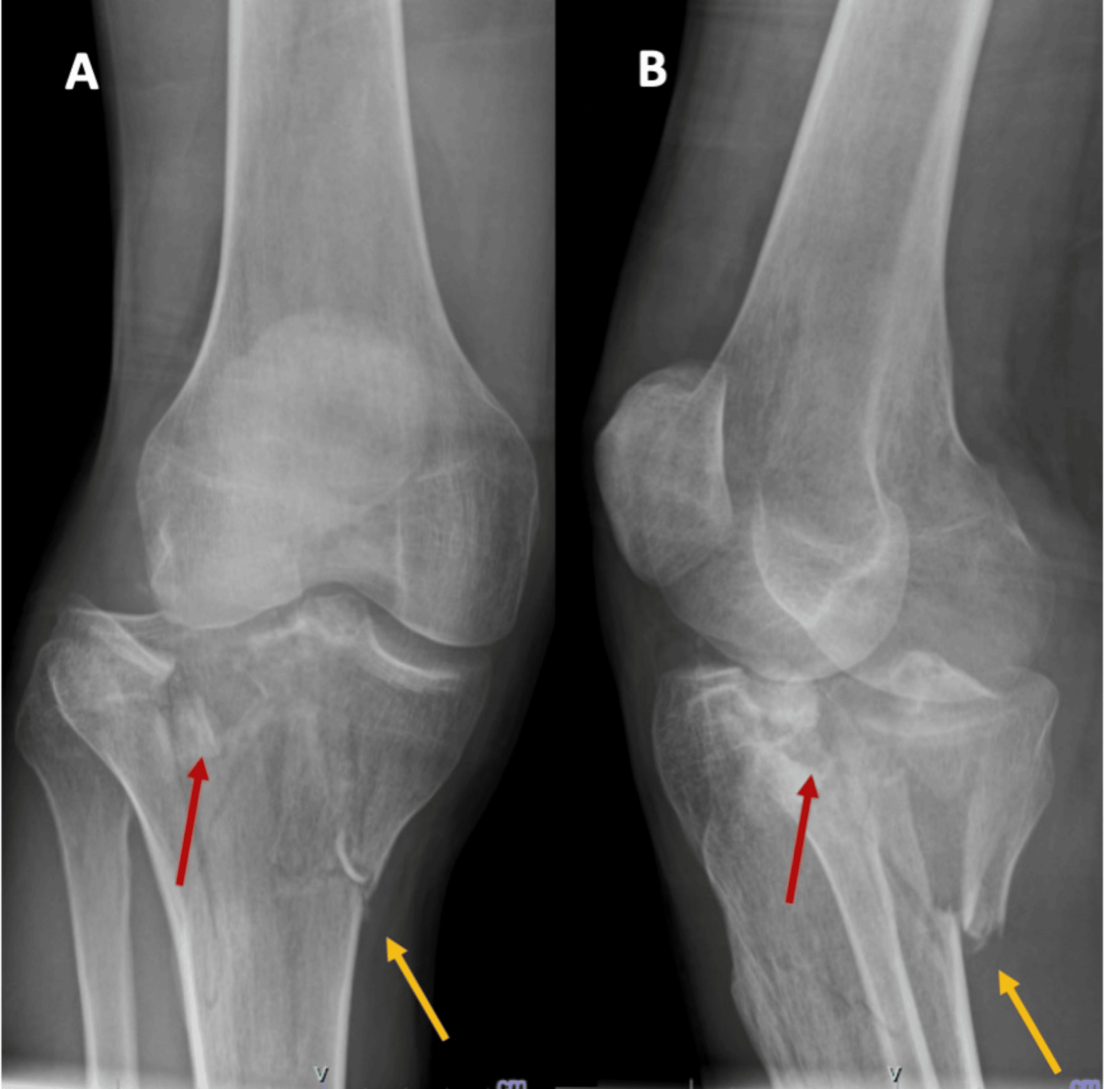
Preoperative radiographs of Patient 1 Radiographs showing a bicondylar tibial plateau fracture (Schatzker V), with depression of the lateral tibial plateau (red arrows) and a split of the medial tibial plateau (yellow arrows), in both the AP view (A) and the oblique view (B).

**Figure 2:**
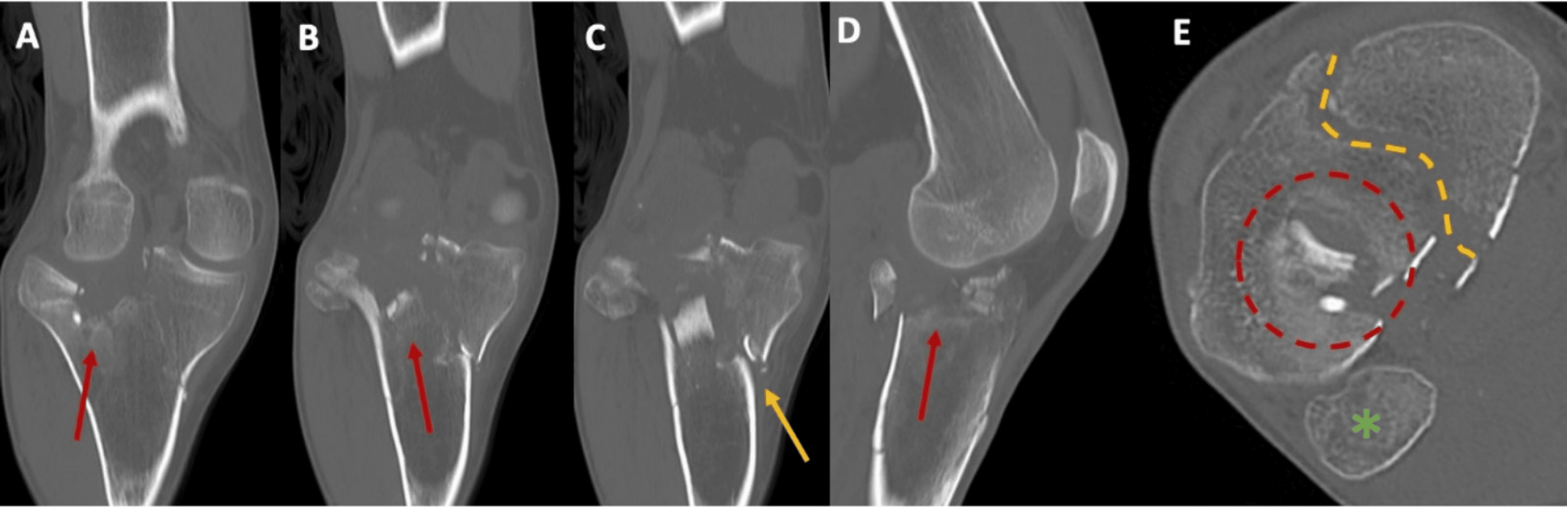
Preoperative CT scans of Patient 1 Coronal views (A-C) show a bicondylar tibial plateau fracture (Schatzker V), with depression of the lateral tibial plateau (red arrows) and a split of the medial tibial plateau (yellow arrow). Sagittal view (D) displays the depression fragment (red arrow). Axial view (E) highlights the fibular head (green asterisk) as a reference point for the lateral and medial sides, with the depression fragment (circular red dotted line) and split fracture line (yellow dotted line) outlined.

**Figure 3:**
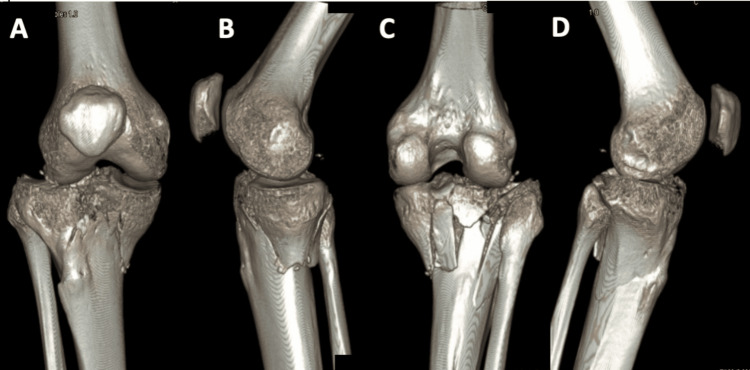
Preoperative CT scan 3D reconstructions of Patient 1 The 3D reconstructions from the CT scan provide detailed visualization of the fracture pattern on the anterior (A), medial (B), posterior (C), and lateral (D) sides.

Patient 1, a 35-year-old male, was involved in a motor vehicle accident and presented with a Schatzker type V tibial plateau fracture of the right knee, as demonstrated in the initial radiographs (AP and lateral views) and CT scan images shown in Figure 1, Figure 2, and Figure 3.

**Figure 4:**
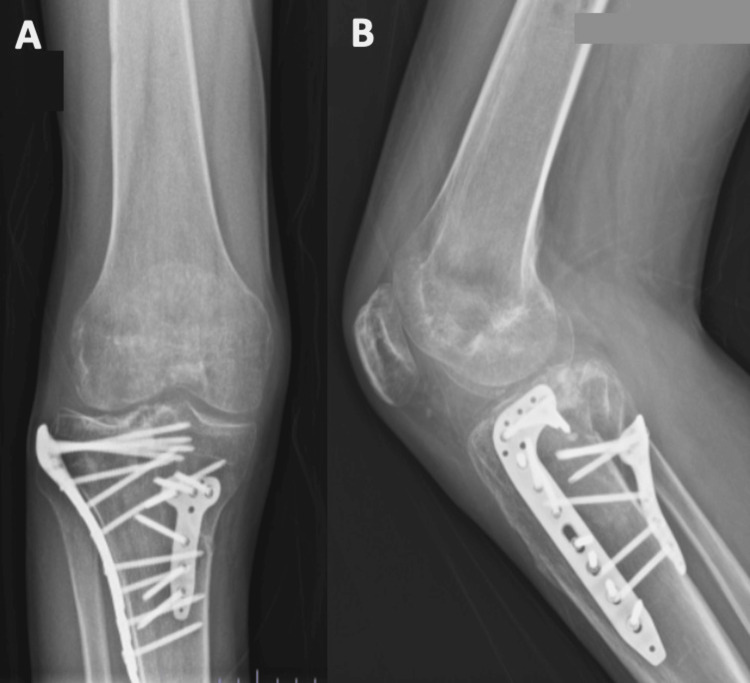
Postoperative radiographs of Patient 1 at three months The radiographs demonstrate the positioning of the plates on the anterolateral and posteromedial sides, shown in both the AP (A) and lateral (B) views. The depressed fragment has been reduced and is being supported by the bone graft.

This patient was treated using an anterolateral and posteromedial approach (Lobenhoffer approach), with double plating, and a tricalcium phosphate bone graft was used to fill the cancellous bone defect. During surgery, the patient exhibited a slight residual articular step-off of the lateral tibial plateau, which could not be corrected due to the initial fracture displacement and fragmentation (Figure 4).

**Figure 5:**
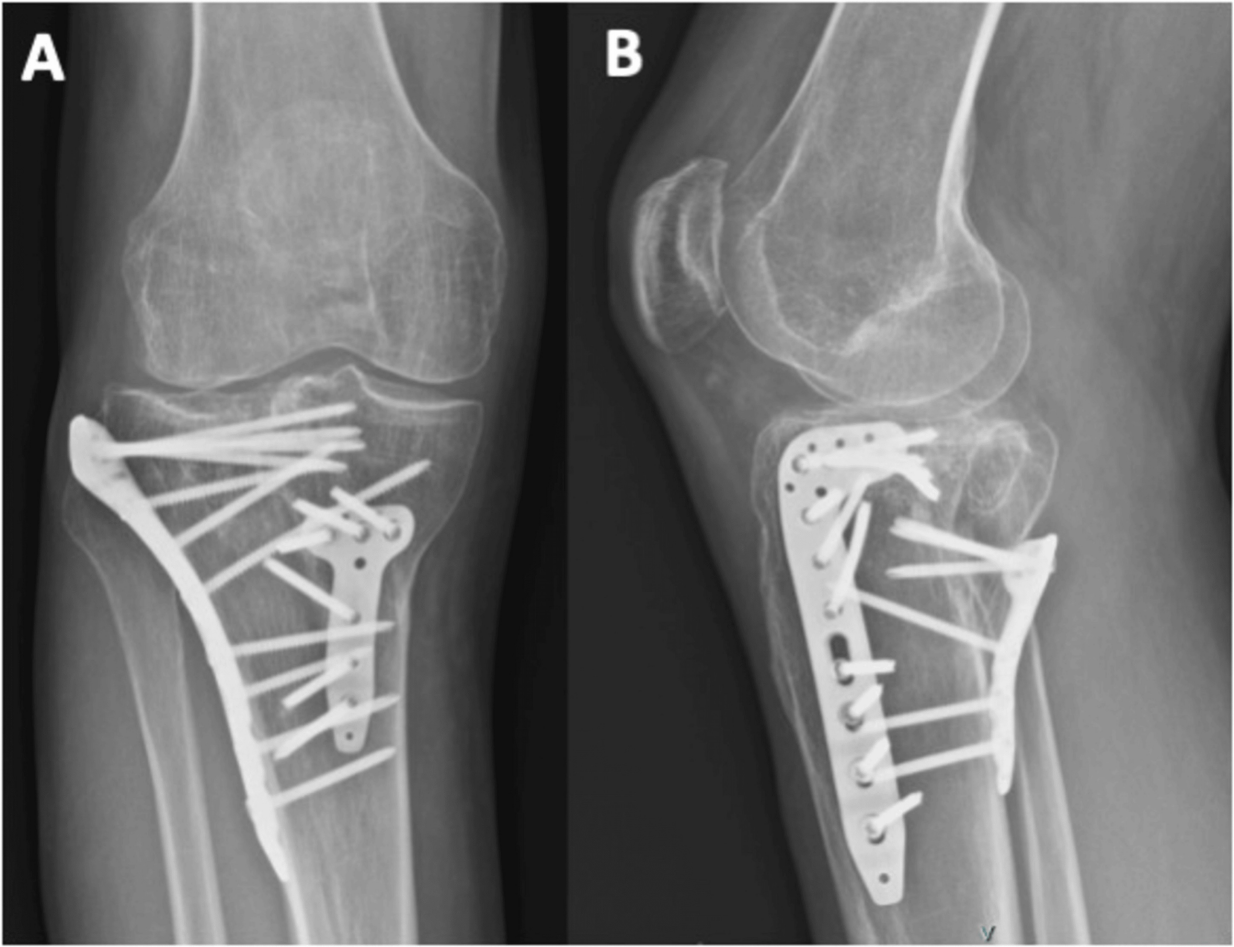
Postoperative radiographs of Patient 1 at 18 months The radiographs show no significant changes in articular congruency compared to those taken at three months postoperative. There is clear evidence of fracture healing and successful integration of the bone graft.

At 18 months postoperatively, no significant changes were observed in articular congruency, as shown in Figure 5.

**Figure 6:**
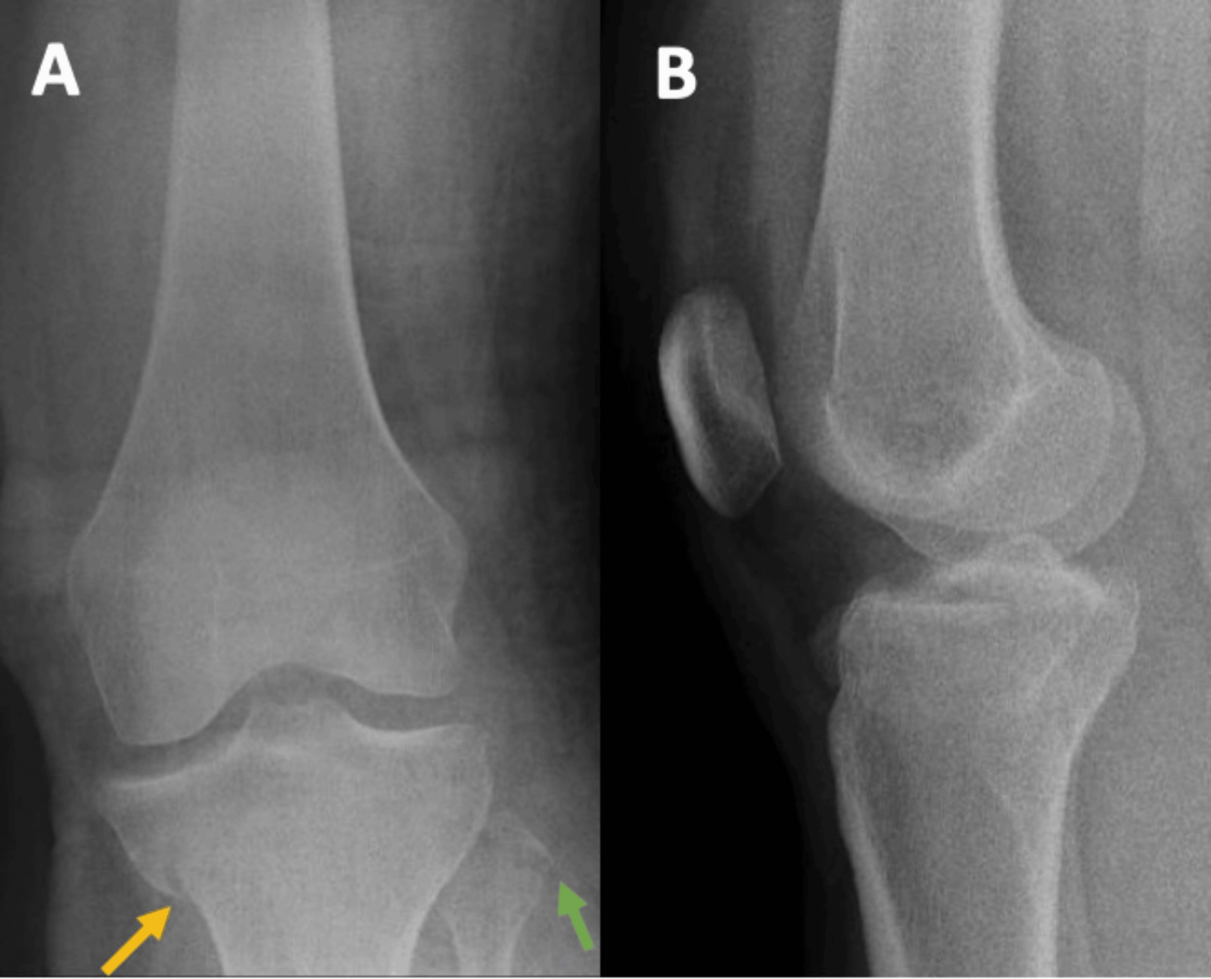
Preoperative radiographs of Patient 2 In both the AP (A) and lateral (B) views, the radiographs reveal what appears to be a simple split fracture of the tibia at the medial plateau (yellow arrow). However, the presence of a fibular head fracture (green arrow) suggests the possibility of a more complex injury.

**Figure 7:**
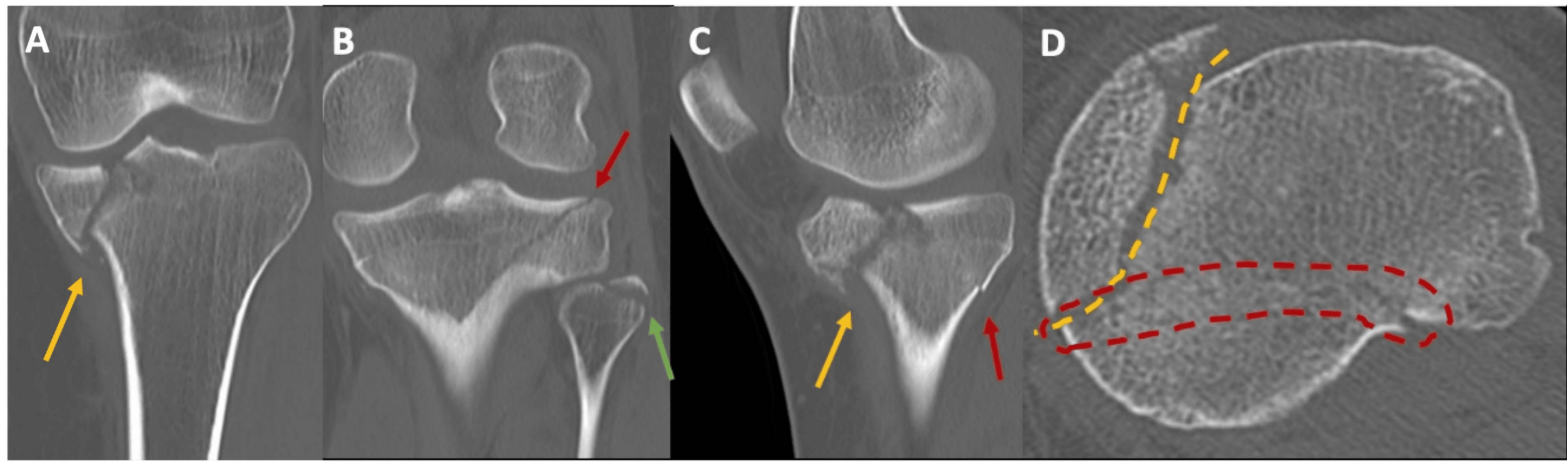
Preoperative CT scans of Patient 2 The coronal views from the CT scans (A, B) show a medial tibial plateau fracture (yellow arrow), a fibular head fracture (green arrow), and a fracture line extending to the lateral tibial plateau (red arrow). The sagittal view (C) displays the medial plateau fracture line (yellow arrow) and a coronal plane fracture line (red arrow). In the axial view (E), the medial fracture line is outlined by the yellow dotted line, and the coronal plane fracture is outlined by the oval-shaped red dotted line.

**Figure 8:**
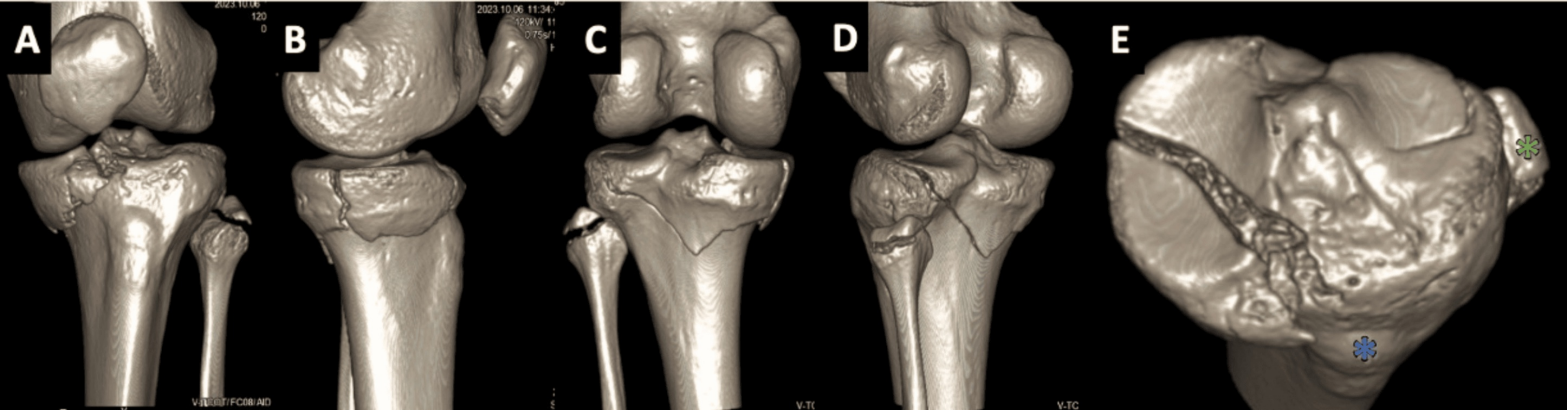
Preoperative CT scan 3D reconstructions of Patient 2 The 3D reconstructions from the CT scan provide a detailed view of the fracture pattern from several angles: anterior (A), medial (B), posterior (C), and posterolateral (D). Additionally, the axial view (E) offers further details, highlighting the fibular head (green asterisk) and the anterior tibial tuberosity (blue asterisk) for spatial reference.

Patient 2, a 22-year-old female, was also involved in a motor vehicle accident and presented with a Schatzker type V tibial plateau fracture of the left knee, with the distinctive feature of a coronal plane fracture. This can be seen in the initial radiographs (AP and lateral views) and CT scan images shown in Figure 6, Figure 7, and Figure 8.

**Figure 9:**
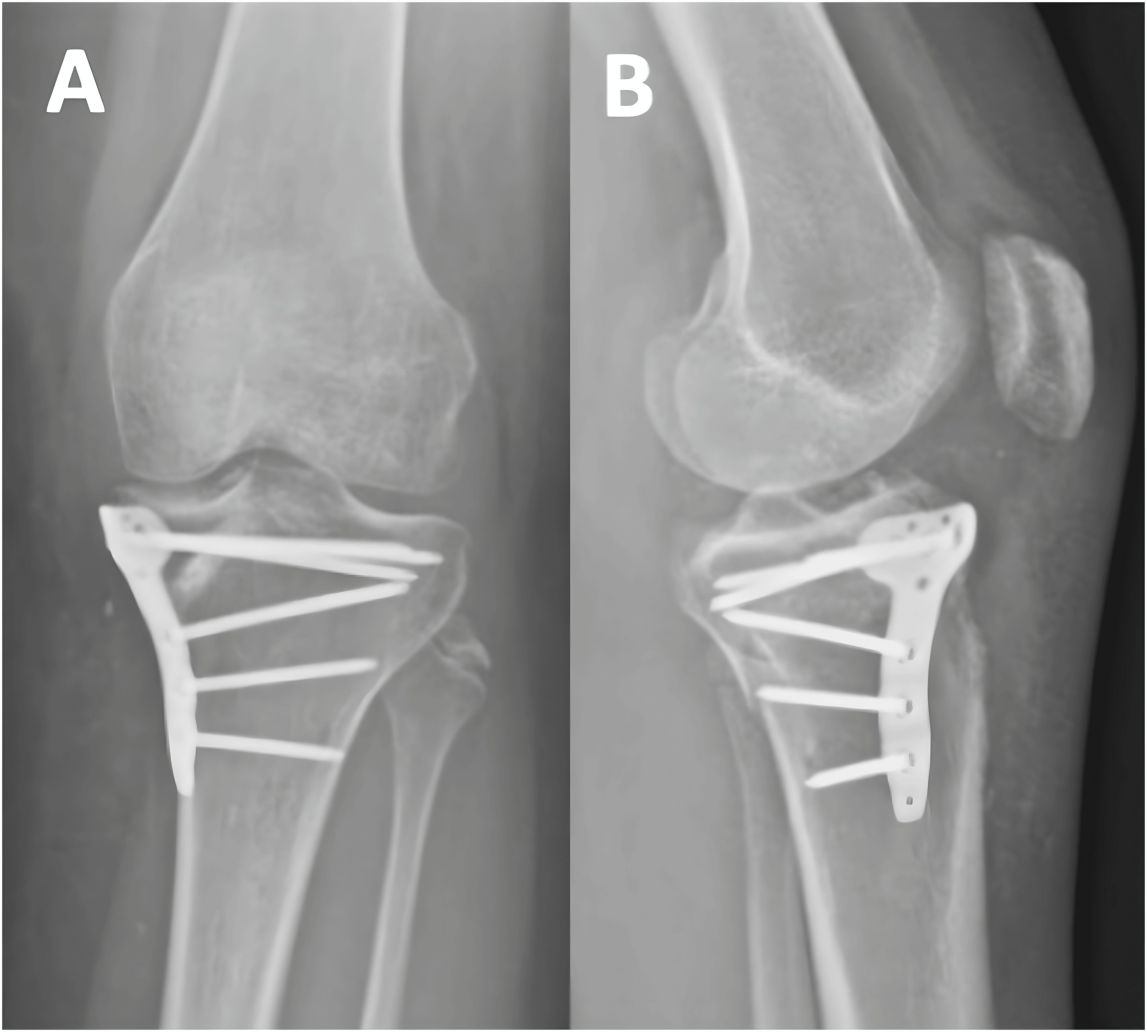
Postoperative radiographs of Patient 2 at three months The radiographs illustrate the positioning of the plate on the anteromedial side, as seen in both the AP (A) and lateral (B) views, with the bone graft visible as a radiopaque line on the medial side.

This patient was treated using an anteromedial approach with a buttress plate, and tricalcium phosphate bone graft was used to fill the cancellous bone defect, as shown in the end result in Figure 9.

**Figure 10:**
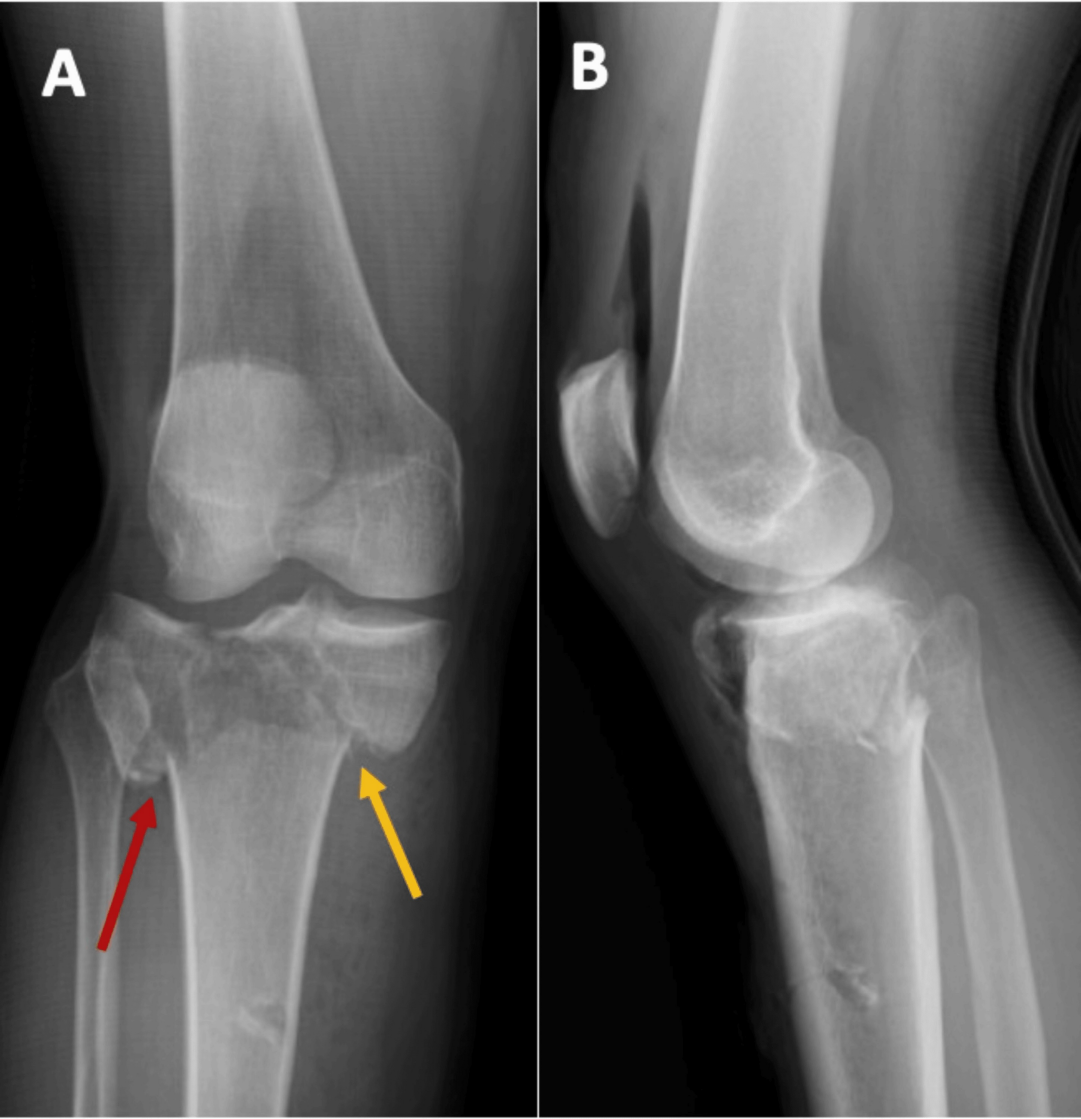
Preoperative radiographs of Patient 3 The radiographs show a bicondylar tibial plateau fracture (Schatzker V) in both the AP view (A) and the lateral view (B). The lateral tibial plateau fracture is outlined by the red arrow, and the medial tibial plateau fracture line is outlined by the yellow arrow in the AP view (A).

**Figure 11:**
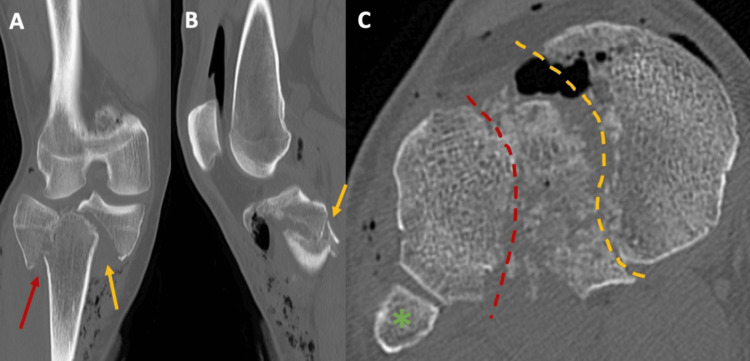
Preoperative CT scans of Patient 3 The coronal CT scan view (A) shows the bicondylar tibial plateau fracture (Schatzker V), with the split fracture of the lateral tibial plateau (red arrow) and the split fracture of the medial tibial plateau (yellow arrow) clearly visible. The sagittal CT scan view (B) highlights details of the medial tibial plateau fracture (yellow arrow). In the axial view (E), the fibular head (green asterisk) serves as a reference point for the lateral and medial sides, with both split fractures of the lateral (red dotted line) and medial plateau (yellow dotted line) outlined. Comminution between the two larger fragments, lateral and medial, is also visible.

**Figure 12:**
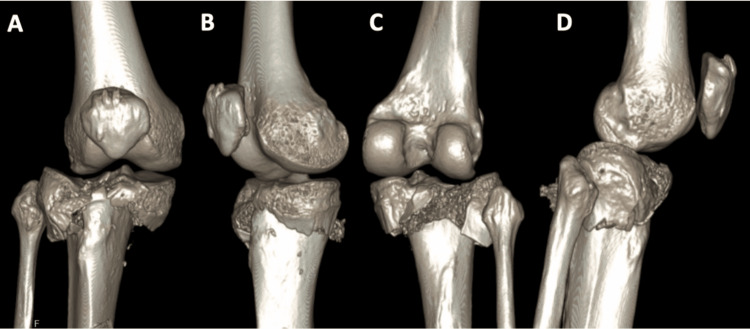
Preoperative CT scan 3D reconstructions of Patient 3 The CT scan 3D reconstructions provide detailed visualization of the fracture pattern from the anterior (A), medial (B), posterior (C), and lateral (D) sides.

Patient 3, a 56-year-old male, was involved in a motorcycle accident and presented with a Schatzker type V tibial plateau open fracture (Gustilo-Anderson Type I) of the right knee, as shown in the initial radiographs (AP and lateral views) and CT scan images in Figure 10, Figure 11, and Figure 12.

**Figure 13:**
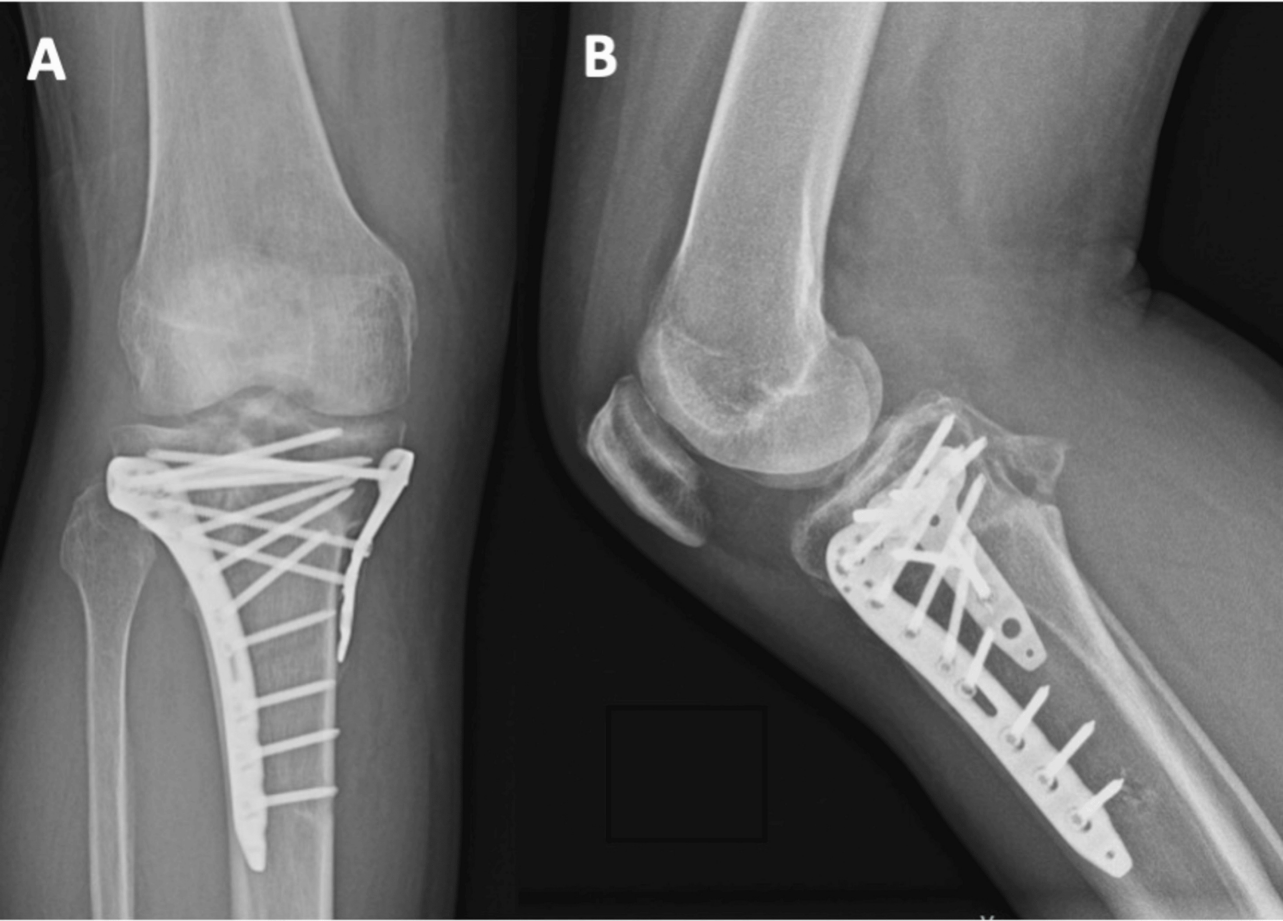
Postoperative radiographs of Patient 3 at three months The radiographs, in both AP (A) and lateral (B) views, show a successful reduction of the articular surface. Both the anterolateral and medial plates, along with the bone graft, provide support to the original fracture.

This patient was treated using an anterolateral and medial approach, with ORIF using double plating, and tricalcium phosphate bone graft was used to fill the cancellous bone defect, as shown in the end result in Figure 13.

Surgical technique and follow-up

Patient 3 was promptly taken to the operating room due to an exposed fracture, while Patients 1 and 2 underwent surgery within 72 hours, during which time immobilization with a lower leg cast was applied. All surgeries were performed under spinal anesthesia, combined with epidural anesthesia. The patients were positioned supine on the operating table, with a tourniquet applied to the thigh of the affected lower limb. Dual incisions were made for Patients 1 and 3, with anterolateral and medial/posteromedial approaches. For Patient 2, only one incision was made through an anterolateral approach.

To ensure adequate exposure of the tibial plateau, once proper access to the fracture site was achieved, a submeniscal arthrotomy was performed to assess the articular surface and facilitate anatomic reduction with direct visual control. The depressed fragments were elevated, and the articular surface was anatomically reduced, with reduction confirmed both visually and under fluoroscopy. The reduction was temporarily maintained with Kirschner wires. Through the fracture line, which was opened using an osteotome, the cancellous bone defect was filled with a tricalcium phosphate bone graft.

**Figure 14:**
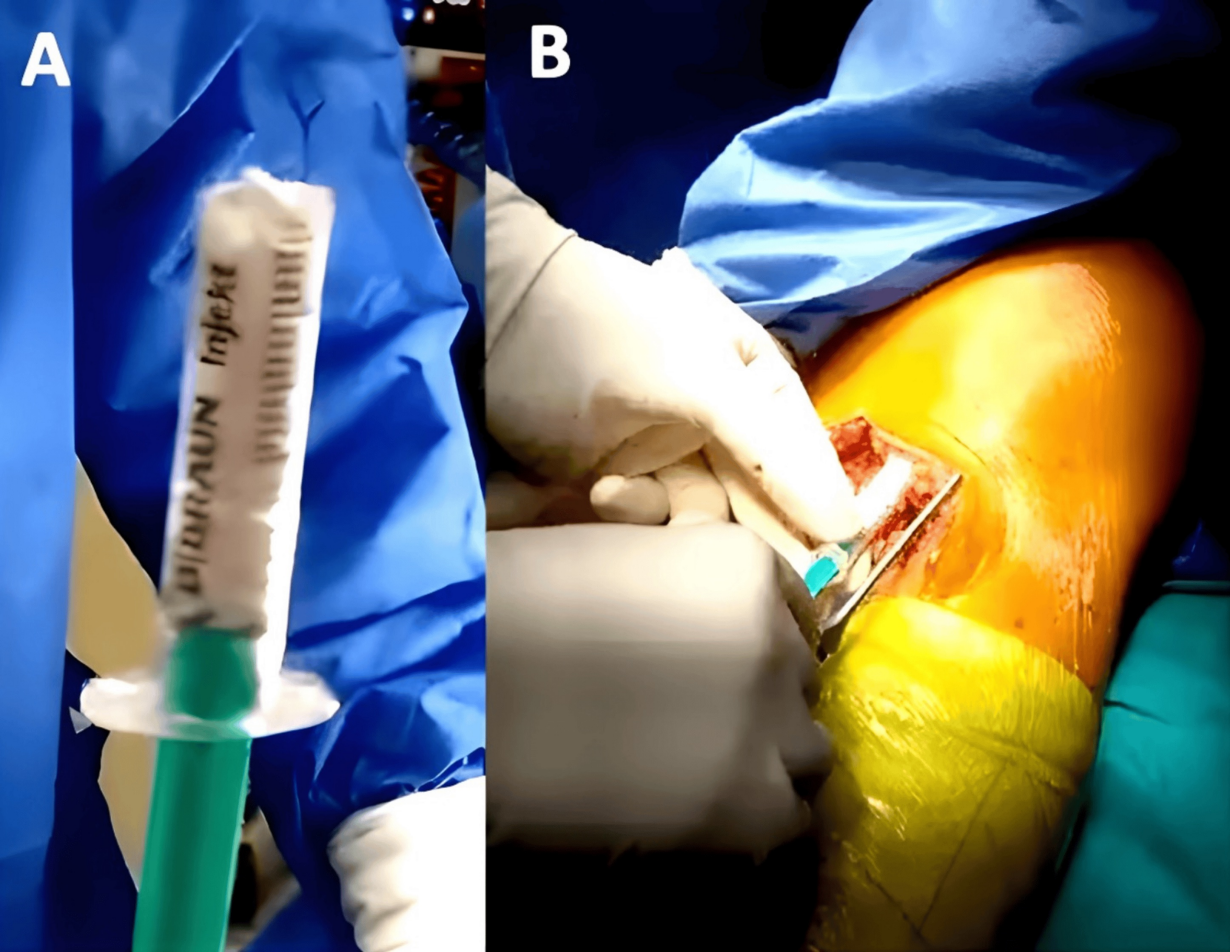
Method of application of the tricalcium phosphate bone filler (AdvanCore®) In the left image (A), a syringe containing bone graft granules has had its top cut off to facilitate easier graft application. In the right image (B), the syringe is being used to compact and position the graft at the fracture site.

AdvanCore^®^ is available in the form of blocks, wedges, and granules. In all three patients, we chose to use the granules of tricalcium phosphate bone filler, which were applied using a syringe with the top cut off to aid in compacting the graft, as shown in Figure 14.

Fluoroscopy was used to ensure the correct application of the graft and proper filling of the defect. Surgical approaches and proximal tibial plates were chosen based on the fracture lines. Fixation was achieved using appropriate screws, and final reduction and fixation were again verified under fluoroscopy. The submeniscal arthrotomy was then closed, followed by layered closure of the surgical wound.

All patients began physical therapy with early range of motion (ROM) exercises. Partial weight-bearing was permitted after six weeks post-surgery, and full weight-bearing was initiated at eight weeks or once fracture healing was confirmed radiologically, depending on the surgeon’s preference for postoperative management.

Postoperative pain was managed with analgesic medication. Physical therapy continued at our outpatient clinic, with follow-up assessments at two and six weeks, as well as at three, six, and 12 months. Plain radiographs (AP and lateral views) were obtained during follow-up to monitor bone consolidation and to check for any loss of reduction or articular depression.

Results

Three patients were included in this study: two males and one female, with a median age of 35 years. All patients presented with a Schatzker type V fracture, with one patient (Patient 2) having a particularly complex fracture in the coronal plane. In Patients 1 and 3, fixation was achieved using two plates through anterolateral and medial/posteromedial approaches. In Patient 2, the fracture was fixed with one plate on the anteromedial side. No intraoperative complications were reported. Patient demographics, fracture details, and surgical descriptions are shown in Table 1.

**Table 1:** Patient demographics, fracture details, and surgical description

Patient no.	Age (years)	Sex	Fracture laterality	Schatzker classification	Three-column concept classification	Fixation method
1	35	M	Right	V	Three columns	Two plates (anterolateral and posteromedial)
2	22	F	Left	V (with coronal plane fracture)	Two columns (anteromedial and posterior)	Anteromedial plate
3	56	M	Right	V	Three columns	Two plates (anterolateral and medial)

The median follow-up time was eight months, and no complications were observed during this period. Patient 1 exhibited a slight residual articular step-off of the lateral tibial plateau intraoperatively, which did not worsen during the follow-up period. Patient follow-up time, knee ROM, complications, and test scores are presented in Table 2.

**Table 2:** Patient follow-up time, ROM, complications, and test scores OKS: Oxford Knee Score; ROM: range of motion; VAS: Visual Analog Scale

Patient no.	Follow-up time (months)	ROM (degrees)	Tibial plateau depression during follow-up	Complications	Patient satisfaction (0-10)	VAS for pain (0-10)	OKS (0-48)	EQ-5D-5L values	EQ-5D-5L VAS (0-100)
1	18	0-110º	No	0	10	4	37	21221	95
2	8	0-120º	No	0	10	0	37	11112	100
3	3	0-90º	No	0	6	4	22	33332	70

The mean values for patient-reported scores are displayed in Table 3.

**Table 3:** Mean values for patient-reported scores OKS: Oxford Knee Score; VAS: Visual Analog Scale

Evaluated variable	Mean (±SD) patient-reported score
Patient satisfaction (0-10)	8.67 (±2.31)
VAS for pain (0-10)	2.67 (±2.31)
OKS (0-48)	32 (±8.66)
EQ-5D-5L VAS (0-100)	88.33 (±16.07)

Patient 1 resumed work three months postoperatively, Patient 2 resumed her university studies three months postoperatively, and Patient 3 returned to work four months after surgery.

## Discussion

Tibial plateau fractures are typically associated with a high rate of complications and loss of fixation, primarily due to the high degree of articular comminution and bone voids in the metaphyseal area. Ali et al. reported an overall 31% rate of fixation failure in tibial plateau fractures, with 79% in patients over 60 years of age and 7% in younger patients [3]. The study also identified several factors statistically associated with loss of reduction, including age over 60 years, premature weight-bearing, preoperative displacement, fracture fragmentation, and severe osteoporosis [3]. After the reduction of depressed tibial plateau fractures, bone grafting may be necessary to restore the structural integrity of the defect [11].

Bone grafting is effective in preventing loss of reduction and failure of internal fixation while contributing significantly to joint stability [5,12]. Autograft is considered the gold standard due to its osteoinductive growth factors, osteogenic cells, and osteoconductive scaffold [13]. However, the use of autografts is associated with donor site morbidity and limited graft availability [11]. Allograft offers a viable alternative to autograft, although concerns regarding supply logistics and the potential risk of disease transmission remain [14].

In recent years, bone graft substitutes have gained popularity, particularly in trauma cases. Synthetic materials, such as calcium phosphates and apatite calcium sulfates, are among the most commonly used. Several studies have demonstrated the advantages of these bone void fillers, particularly in the treatment of tibial plateau fractures [5,11,15,16]. Lobenhoffer et al. found that injectable calcium phosphate bone cement used as a graft exhibited slow degradation and reabsorption, with its integration taking longer [17]. Consequently, tricalcium phosphate and calcium phosphate ceramics with porous structures, which enhance graft reabsorption, are preferred [5]. The porosity of tricalcium phosphate improves blood diffusion, enhancing cell-mediated reabsorption and the local metabolic rate [5].

In a study by Shen et al., tricalcium phosphate was used in the treatment of 124 patients with depressed tibial plateau fractures, yielding good to excellent results in 95.2% of patients, as assessed using the Hospital for Special Surgery knee score [18]. No osteoarthritic signs or loss of reduction were observed on radiographs [18]. Full weight-bearing was restricted until fracture healing was confirmed radiologically, although partial weight-bearing was allowed after six weeks [18]. Welch et al. reported that the use of calcium phosphate cement prevented subsidence of fracture fragments and maintained articular congruency [19].

A comparative study by Russel et al. enrolled 120 patients with unstable tibial plateau fractures, treating 82 with calcium phosphate cement and 38 with autologous grafts. The study found a higher rate of articular subsidence in the bone graft group, which was statistically significant [20]. Yetkinler et al. suggested that the non-weight-bearing postoperative period could be significantly reduced by using calcium phosphate cement to fill the void created by depressed tibial plateau fractures, as it was associated with less articular collapse [12].

In our series, although all fractures were classified as Schatzker type V, the severity varied, necessitating individualized treatment plans and surgical approaches. Patients 1 and 3 had more severe cases, with significant articular depression and displacement. For these patients, ORIF with two plates was the treatment of choice. The surgical approaches were based on fracture line orientation, with both patients requiring an anterolateral approach, combined with a posteromedial approach for Patient 1 and a medial approach for Patient 3. Patient 2, however, presented with a unique fracture pattern, characterized by mild depression of the medial tibial plateau and a fracture line in the coronal plane. ORIF was performed with a single plate, as the posterior column showed minimal displacement, and the anteromedial plate allowed for optimal screw placement to support the posterior column.

We obtained good clinical and functional results, as well as high levels of patient satisfaction. Although Patient 1 exhibited a slight residual articular step-off of the lateral tibial plateau during surgery that could not be corrected due to the initial fracture displacement and fragmentation, the articular surface did not collapse further during the follow-up period, likely due to the support provided by the bone void filler.

The use of tricalcium phosphate bone graft, with its chemical composition similar to the human bone mineral phase and its interconnected porosity that allows for complete vascularization, enables steady osteointegration, and promotes faster recovery, as reported in similar studies [5,18].

This study has some limitations, including its retrospective design and small sample size. Additionally, the relatively short follow-up period may conceal potential complications that could arise in the future, especially in Patient 3, who had not yet begun full weight-bearing at the time of this study. The short follow-up for Patient 3 may also explain the lower OKS and EQ-5D-5L scores observed.

## Conclusions

Tricalcium phosphate bone grafts offer several advantages when used to support the internal fixation of comminuted and depressed tibial plateau fractures. Along with other synthetic bone void fillers, they present a practical alternative to autografts and allografts, as they are readily available, providing an almost unlimited supply of material while avoiding the morbidity associated with bone graft harvesting. Additionally, tricalcium phosphate bone grafts offer mechanical support and facilitate vascular ingrowth, which is linked to favorable osteointegration rates.

Overall, our case series demonstrates promising results with the use of tricalcium phosphate bone grafts in treating comminuted and depressed tibial plateau fractures, positioning this material as a viable and safe alternative to autografts and allografts in similar cases. However, further research with prospective study designs and larger sample sizes is necessary to validate these findings and evaluate long-term clinical and radiological outcomes.

## References

[1] Giannoudis P, Einhorn T, Marsh D (2007). Fracture healing: the diamond concept. Injury.

[2] Nicholson JA, Makaram N, Simpson A, Keating JF (2021). Fracture nonunion in long bones: a literature review of risk factors and surgical management. Injury.

[3] Ali AM, El-Shafie M, Willett KM (2002). Failure of fixation of tibial plateau fractures. J Orthop Trauma.

[4] Ali AM, Saleh M, Eastell R, Wigderowitz CA, Rigby AS, Yang L (2006). Influence of bone quality on the strength of internal and external fixation of tibial plateau fractures. J Orthop Res.

[5] Oztürkmen Y, Caniklioğlu M, Karamehmetoğlu M, Sükür E (2010). Calcium phosphate cement augmentation in the treatment of depressed tibial plateau fractures with open reduction and internal fixation. Acta Orthop Traumatol Turc.

[6] Rodham P, Giannoudis PV (2022). Innovations in orthopaedic trauma: top advancements of the past two decades and predictions for the next two. Injury.

[7] Oliver RA, Lovric V, Christou C, Walsh WR (2020). Comparative osteoconductivity of bone void fillers with antibiotics in a critical size bone defect model. J Mater Sci Mater Med.

[8] Gonçalves RS, Tomás AM, Martins DI (2012). Cross-cultural adaptation and validation of the Portuguese version of the Oxford Knee Score (OKS). Knee.

[9] Ferreira PL, Ferreira LN, Pereira LN (2013). Contribution for the Validation of the Portuguese version of EQ-5D. Acta Med Port.

[10] Ferreira PL, Pereira LN, Antunes P, Ferreira LN (2023). EQ-5D-5L Portuguese population norms. Eur J Health Econ.

[11] Iundusi R, Gasbarra E, D'Arienzo M, Piccioli A, Tarantino U (2015). Augmentation of tibial plateau fractures with an injectable bone substitute: CERAMENT™. Three year follow-up from a prospective study. BMC Musculoskelet Disord.

[12] Yetkinler DN, McClellan RT, Reindel ES, Carter D, Poser RD (2001). Biomechanical comparison of conventional open reduction and internal fixation versus calcium phosphate cement fixation of a central depressed tibial plateau fracture. J Orthop Trauma.

[13] Faour O, Dimitriou R, Cousins CA, Giannoudis PV (2011). The use of bone graft substitutes in large cancellous voids: any specific needs?. Injury.

[14] Ong JC, Kennedy MT, Mitra A, Harty JA (2012). Fixation of tibial plateau fractures with synthetic bone graft versus natural bone graft: a comparison study. Ir J Med Sci.

[15] Bajammal SS, Zlowodzki M, Lelwica A (2008). The use of calcium phosphate bone cement in fracture treatment: a meta-analysis of randomized trials. J Bone Joint Surg Am.

[16] Simpson D, Keating J (2004). Outcome of tibial plateau fractures managed with calcium phosphate cement. Injury.

[17] Lobenhoffer P, Gerich T, Witte F, Tscherne H (2002). Use of an injectable calcium phosphate bone cement in the treatment of tibial plateau fractures: a prospective study of twenty-six cases with twenty-month mean follow-up. J Orthop Trauma.

[18] Shen C, Ma J, Chen XD, Dai LY (2009). The use of beta-TCP in the surgical treatment of tibial plateau fractures. Knee Surg Sports Traumatol Arthrosc.

[19] Welch RD, Zhang H, Bronson DG (2003). Experimental tibial plateau fractures augmented with calcium phosphate cement or autologous bone graft. J Bone Joint Surg Am.

[20] Russell TA, Leighton RK (2008). Comparison of autogenous bone graft and endothermic calcium phosphate cement for defect augmentation in tibial plateau fractures. A multicenter, prospective, randomized study. J Bone Joint Surg Am.

